# LncRNA FENDRR in Carcinoma-Associated Fibroblasts Regulates the Angiogenesis of Oral Squamous Cell Carcinoma Through the PI3K/AKT Pathway

**DOI:** 10.3389/fonc.2021.616576

**Published:** 2021-07-13

**Authors:** Yuming Xu, Erhui Jiang, Zhe Shao, Zhengjun Shang

**Affiliations:** ^1^ The State Key Laboratory Breeding Base of Basic Science of Stomatology (Hubei-MOST) & Key Laboratory of Oral Biomedicine Ministry of Education, School & Hospital of Stomatology, Wuhan University, Wuhan, China; ^2^ Department of Oral and Maxillofacial Head Neck Surgery, School & Hospital of Stomatology, Wuhan University, Wuhan, China

**Keywords:** LncRNA, angiogenesis, OSCC, PI3K - AKT pathway, CAFs, FENDRR

## Abstract

Angiogenesis is essential for the development of tumors. Studies have shown that carcinoma-associated fibroblasts (CAFs) are involved in regulating tumor angiogenesis, but the mechanism remains unclear. Recently, long noncoding RNAs (lncRNAs) have been proved to play an important role in the angiogenesis of various tumors. However, there is currently no research involving the regulation of CAFs on the angiogenesis of oral squamous cell carcinoma (OSCC) mediated by lncRNAs. By analyzing microarray data, we identified that the expression of lncRNA FOXF1 adjacent noncoding developmental regulatory RNA (FENDRR) in OSCC patients is downregulated, compared to that in normal tissues. Quantitative polymerase chain reaction (qPCR) results demonstrated that FENDRR expression is lower in CAFs compared to normal fibroblasts (NFs) of OSCC patients. KEGG pathway analysis revealed that some genes differentially expressed between CAFs and NFs of HNSCC patients are enriched to the PI3K/AKT pathway. Further experiments confirmed that the downregulation of FENDRR can activate the PI3K/AKT pathway in NFs and enhances the expression of matrix metalloproteinase 9 (MMP9). The overexpression of FENDRR had the opposite effect. Besides, we co-cultured human umbilical vein endothelial cells (HUVECs) with CAFs, and the tube-forming ability of HUVECs co-cultured with CAFs overexpressing FENDRR decreased significantly. However, activation of the AKT pathway of CAFs overexpressing FENDRR can weaken the inhibitory effect of FENDRR on angiogenesis. In summary, our experiments are focused on the influence of lncRNAs in CAFs on OSCC angiogenesis for the first time and prove that FENDRR mediates CAFs’ regulation of OSCC angiogenesis through the PI3K/AKT pathway.

## Introduction

Oral squamous cell carcinoma (OSCC) is the most common type of head and neck squamous cell carcinoma (HNSCC) which ranks sixth among cancers worldwide (1). The prognosis of patients with OSCC is poor, especially for metastatic patients, whose 5-year survival rate is only about 40% (2). Angiogenesis is a critical factor leading to the low survival rate of OSCC patients, which has been illustrated to be closely related to the metastasis of OSCC (3, 4). The formation of new blood vessels around the tumor facilitates the tumor’s access to oxygen and nutrients. Besides, the increased permeability of newly formed blood vessels is conducive to the metastasis of tumor cells (5, 6). Therefore, controlling angiogenesis plays an important role in the fight against cancer.

Carcinoma-associated fibroblasts (CAFs), often known as activated fibroblasts, are generally derived from the tissue-resident normal fibroblasts (NFs) and can promote tumor angiogenesis by secreting higher levels of proteolytic enzymes, such as matrix metalloproteinase 9 (MMP9) (7). Recently, The proangiogenic effect of CAFs has been demonstrated in various cancers, such as breast cancer (8), melanoma (9), and colorectal cancer (10). However, few studies have addressed the role of CAFs in OSCC angiogenesis.

Long noncoding RNAs (lncRNAs) are a subtype of RNA with a length of more than 200 nucleotides that have little or no coding potential (11). To date, lncRNAs have been found to be abnormally expressed in various cancers, such as colon cancer (12), gastric cancer (13), and cervical cancer (14), and regulate tumor angiogenesis through different mechanisms. In addition, some lncRNAs were demonstrated to be differentially expressed in OSCC (15), regulate OSCC development (16), and can be used as prognostic biomarkers for OSCC (17). Moreover, studies have confirmed that the abnormal expression of lncRNAs has a non-negligible effect on the biological function of CAFs (18, 19). However, there has been no research involving the role of lncRNAs in the proangiogenic effect of CAFs derived from OSCC.

Herein, for the first time, we explored the regulation of lncRNAs on the proangiogenic effect of CAFs derived from OSCC. Our experiments show that lncRNA FENDRR, downregulated in CAFs derived from OSCC, can inhibit the proangiogenic effect of CAFs through the PI3K/AKT pathway, which makes FENDRR a potential therapeutic target for anti-angiogenic therapy of OSCC treatment.

## Materials and Methods

### GEO Data Download and Annotation

An OSCC microarray profiling dataset (GSE9844) and a fibroblasts RNA-seq dataset (GSE83314) were downloaded from the GEO database (https://www.ncbi.nlm.nih.gov/geo/query/acc.cgi?acc=GSE9844 and https://www.ncbi.nlm.nih.gov/geo/query/acc.cgi?acc=GSE83314). The “hgu133plus2.db” package was used to perform probe reannotation for the GSE9844 profiling dataset. The “FactoMineR” and “factoextra” packages were used to perform the principal component analysis (PCA). GSE9844 profiling data was used to verify the differential expression of FENDRR in OSCC tissues compared with normal tissues. The “ggpubr” package was used to draw statistical charts. The RNA-seq dataset GSE83314 was used to identify differentially expressed genes in CAFs compared with NFs using the “edgeR” package in R software. |Fold Change| > 1 and *P* < 0.05 were set as the statistical threshold of differentially expressed genes.

### KEGG Pathways Enrichment Analysis and Survival Analysis

The “clusterProfiler” package was used to perform the Kyoto Encyclopedia of Genes and Genomes (KEGG) pathways enrichment analysis. Differentially expressed genes in CAFs compared with NFs were used for enrichment analysis. The statistical threshold of enriched pathways was set to *P* < 0.05. The survival analysis was performed using the GEPIA database (http://gepia.cancer-pku.cn/) (20). Quartile was used as the Group Cutoff. *P* < 0.05 was considered to be significant.

### Antibodies and Reagents

MMP9 antibody was obtained from Proteintech (Wuhan, China). β-actin antibody, anti-rabbit IgG, and anti-mouse IgG were acquired from Abbkine (CA, USA). AKT1, phospho-AKT1 (P-AKT1), phosphatidylinositol-3-kinase (PI3K), phospho-PI3K (P-PI3K), and vimentin antibodies were purchased from Cell Signaling Technology (MA, USA). FAP antibody was obtained from Abcam (Cambridge, England). Matrigel used for tube formation assay was acquired from Corning (NY, USA). Bicinchoninic acid protein assay kit used to measure the protein concentration was purchased from Thermo Fisher. Trizol reagent for RNA extraction was obtained from Takara (Kyoto, Japan). SC79 was acquired from TOPSCIENCE (Shanghai, China).

### Cell Culture

The CAFs and NFs were isolated from 7 OSCC patients according to a previous study (21). To put it simply, the OSCC tissues and normal tissues were cut into 1-2 mm size tissue blocks, and then put into a 15 ml centrifuge tube and washed with PBS (phosphate buffered saline) containing 5% double antibiotics (Penicillin and Streptomycin) for 8 times. Subsequently, the tissue blocks were inoculated to the bottom of the culture bottle and put into a constant temperature cell incubator. After 4-6 hours, 1 ml of complete medium containing 20% FBS was added. When it was observed that the cells crawling out between different tissue blocks come into contact, cell passage can be carried out. All procedures conformed with the Ethics Committee of School and Hospital of Stomatology at Wuhan University. The OSCC cell lines SCC25 and CAL27 were purchased from the China Center for Type Culture Collection (Shanghai, China). The OSCC cell lines SCC4, SCC9, and HN4 were kindly provided by Doctor Xinmiao‐Wang. The fibroblasts, HN4 and CAL27 were cultured in high-glucose Dulbecco’s modified Eagle’s medium (DMEM) (HyClone, UT, USA) containing 10% fetal bovine serum (FBS) (Natocor, Córdoba, Argentina). The SCC4, SCC9, and SCC25 were cultivated in DMEM:F12 (1:1) containing 10% FBS. The HUVECs were purchased from ScienCell (CA, USA) and cultured in endothelial cell medium (ECM) (ScienCell, CA, USA) containing 5% FBS (ScienCell, CA, USA). The human immortalized oral epithelial cells (HIOECs) were kindly provided by Professor Cheng‐zhang Li and Doctor Zhen‐Zhang and were cultured in KGM gold (Lonza, MD, USA) containing 5% FBS (Lonza, MD, USA). All cells were cultivated in a 5% CO_2_ environment at 37°C.

### Cell Immunofluorescence

On the first day, we seeded CAFs and NFs cells in 24-well plates. When the cell confluence reached 70%-90%, the cells were fixed with paraformaldehyde, washed 3 times with PBS solution, and then blocked with BSA for 1 hour. Finally, the cells were incubated with the primary antibody overnight at 4°C. The next day, the cells were incubated with the second fluorescent antibody for 1 hour. Nuclear staining was performed using DAPI and the images of stained cells were obtained using a Fluorescence microscope (Carl Zeiss, Germany).

### Cell Migration Assay

The co-culture model of CAFs and HUVECs was established using Transwell membrane chambers (24-well plate, 8-μm pore size, 6.5-mm insert) acquired from Corning (NY, USA). The CAFs transfected with FENDRR lentiviral vectors (Lent-FENDRR) or negative control vectors (Lent-NC) were seeded into the lower chambers at a density of 3x10^5^ cells per well, cultured for 24 hours. Subsequently, 2.5x10^5^ HUVECs were seeded in serum-free medium in the upper chambers. After 48 hours of incubation, the cells on the chambers were fixed and stained with crystal violet. Finally, five random fields were selected to count the number of cells traversing to the reverse face.

### *In Situ* Hybridization

*In situ* hybridization (ISH) was performed with digoxigenin-labeled FENDRR probe: 5′-DIG-CAGTGGTTGCTGGGGTTGAAGAGAGGGATGAATA-DIG-3′, following the instructions of the ISH kit purchased from Boster (CA, USA). The slices of 4 pairs of OSCC and normal oral mucosa tissues were treated with conventional dewaxing, rehydration, and then 3% hydrogen peroxide at room temperature for 20 minutes. Then, the slices were digested with pepsin diluted with 3% citric acid at room temperature for 20 minutes to expose RNA fragments. Next, the slices were pre-hybridized for 4 hours and hybridized with FENDRR probe (1.5 ug/ml) overnight. Then the slices were blocked with blocking fluid, followed by incubation with biotinylated mouse anti-digoxigenin and avidin-biotin peroxidase. Finally, a DAB kit (Mxb Bio, China) and hematoxylin were used for visualization. The score used for statistical analyses were calculated as: 1.0*(%Weak positive) + 2.0*(%Positive) + 3.0*(%Strong positive), using Aperio ImageScope (Leica, Germany) (22).

### RNA Isolation and Real-Time PCR

Total RNA was extracted from cells using Trizol reagent, which was subsequently reversely transcribed into cDNA using the PrimeScript RT Reagent Kit (Takara, Kyoto, Japan). Real-time quantitative PCR, conducted on a QuantStudio™ 6 Flex (Life Technologies, USA), was performed by using the SYBR^®^ Premix Ex Taq™ II Kit (Takara Bio). The primers synthesized by Sangon Biotech (Shanghai, China) were as follows: FENDRR (forward 5’-GTGATGCAGTTGCTGGCAAA-3’ and reverse 5’-CAGTTGACTGCAAAGCACCC-3’) and glyceraldehyde-3-phosphate dehydrogenase (GAPDH) (forward 5’-GGAGCGAGATCCCTCCAAAAT-3’ and reverse 5’-GGCTGTTGTCATACTTCTCATGG-3’). RNA expression levels, normalized to that of GAPDH, were calculated using the 2^-△△ Ct^ method.

### Western Blotting Analysis

The total protein of cells was extracted on ice using a mammalian protein extraction reagent (MPER) (Thermo Fisher Scientific) containing protease and phosphatase inhibitors (MilliporeSigma), whose concentration was then measured with a bicinchoninic acid protein assay kit. Next, loading buffer (5x) purchased from Beyotime (Shanghai, China) was added into the protein solutions and heated for 10 minutes at 95°C. 10 ug of protein samples were subjected into 10% SDS-PAGE (60 V, 30 minutes; 120 V, 65 minutes), and then transferred to polyvinylidene fluoride membrane (MilliporeSigma) in the electroblotting buffer (100 V, 53 minutes). Subsequently, at room temperature, the membranes were blocked with 5% nonfat milk in Tris-buffered saline for 1.5 hours and then inoculated with antibodies overnight at 4°C. Finally, the bound antibodies were detected using horseradish peroxidase-conjugated anti-rabbit IgG or anti-mouse IgG.

### Lentivirus Vectors and Short Interfering RNAs

FENDRR lentiviral vectors and negative control vectors were purchased from Hanbio (Shanghai, China). The siRNAs targeting FENDRR (si-FENDRR) and negative control siRNAs (si-NC) were purchased from RiboBio (Guangzhou, China). Before transfection, CAFs or NFs were seeded into 6-well plates and cultured to achieve cell confluency of about 50%. For lentiviral vectors transfection, CAFs were treated with vectors for 24 hours. The mixture in the plates was then replaced with fresh medium. For siRNAs transfection, NFs were treated with siRNAs and transfection reagents for 48 hours. The efficiency of transfection was confirmed by using Real-Time PCR after 48 hours. The target sequences of siRNAs were as follows: si-FENDRR1 (CCAGCCATGTGATTCCAAA), si-FENDRR2 (CCACAGAGCTTCATAGAAT).

### Tube Formation Assay

The co-culture model of CAFs and HUVECs was established using Transwell membrane chambers (24-well plate, 0.4-μm pore size, 6.5-mm insert) acquired from Corning (NY, USA). The CAFs transfected with Lent-FENDRR or Lent-NC were seeded into the upper chambers at a density of 3x10^5^ cells per well, cultured for 24 hours. Subsequently, the HUVECs were seeded into the lower chambers precoated with Matrigel (BD Biosciences, San Jose, CA, USA) at a density of 2.5x10^4^ cells per well. Then, the upper and lower chambers were placed together to co-culture CAFs and HUVECs. After 8 hours of incubation, the images of the capillary-like structure were taken using a microscope (Carl Zeiss, Germany).

### Tumor Xenografts in Nude Mice

The experiment was approved by the Review Board of the Ethics Committee of the Hospital of Stomatology at Wuhan University. A mixture of CAL27 (2 × 10^6^) cells and CAFs (5 × 10^5^) in 50 ul PBS with 50 ul Matrigel was inoculated subcutaneously into the flanks of the female BALB/c nude mice (18–20 g; Beijing Vital River Laboratory Animal Technology Co., Ltd., Beijing, China). After 23 days, the mice were euthanized, and the tumor tissues were excised and analyzed. The tumor volumes were calculated according to the formula (width^2^ × length)/2 (23).

### Statistical Analysis

GraphPad Prism 7.0 was used for statistical analysis, and the statistical significance of the differences between groups was determined by Student’s t-test or Wilcoxon test. The results are expressed as means ± SD. When *P <*0.05, the results were considered to be statistically significant.

## Results

### CAFs and NFs Were Extracted From the Tissues of 7 OSCC Patients

**Figure 1:**
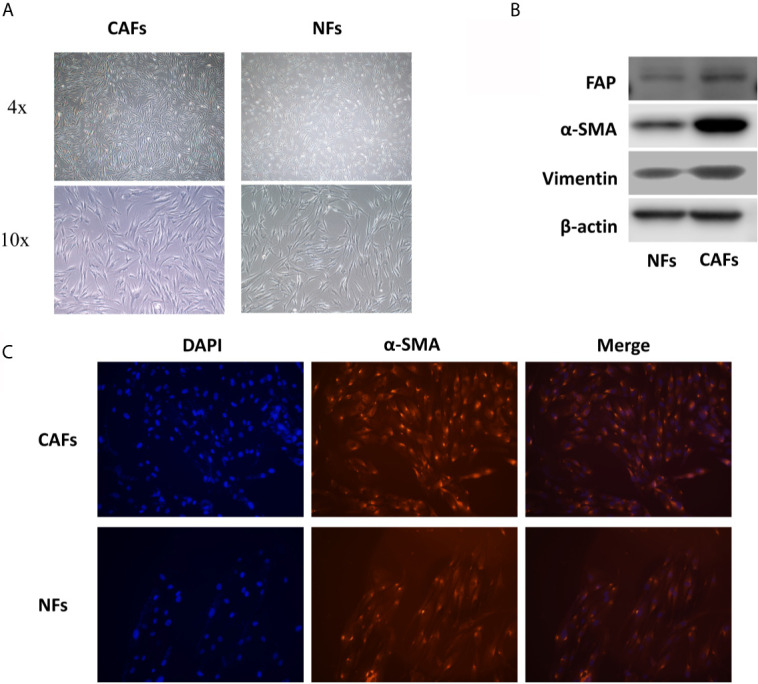
CAFs and NFs were extracted from the tissues of 7 OSCC patients. **(A)** The morphology of CAFs and NFs. **(B)** The results of western blotting of α-SMA, FAP, and vimentin in CAFs and NFs. **(C)** The results of cellular immunofluorescence experiments of α-SMA in CAFs and NFs.

Through the optical microscope, we observed that NFs are mostly slender spindle-shaped and relatively uniform in shape, while CAFs are diverse in shape, mainly short spindle-shaped and polygonal, which accords with the functional diversity of CAFs (**Figure 1A**). Besides, the results of cellular immunofluorescence and western blotting showed that the expression of α-SMA (smooth muscle alpha-actin), vimentin, and FAP (fibroblast activation protein), three markers of CAFs, in CAFs were significantly stronger than that in NFs, which indicated that the primary cells we extracted conformed to the characteristics of CAFs (**Figures 1B, C**).

### FENDRR Is Low Expressed in OSCC Tissues and CAFs Derived From OSCC

**Figure 2:**
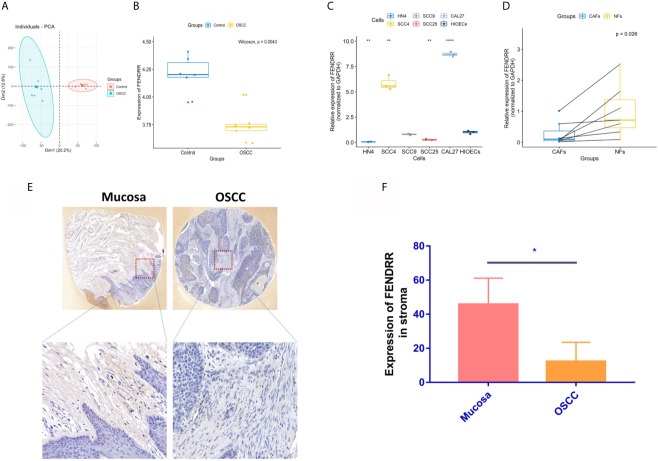
Gene expression analyses of FENDRR. **(A)** 12 groups of data from the GSE9844 dataset were selected through PCA. **(B)** Selected 12 groups were used for differential expression analysis of FENDRR in OSCC and negative control. **(C)** Real-time PCR was used to detect the expression of FENDRR in SCC4, SCC9, SCC25, CAL27, HN4, and HIOECs. **(D)** Real-time PCR was used to detect the expression of FENDRR in 7 pairs of CAFs and NFs derived from OSCC tissues. **(E, F)** The expression of FENDRR in 4 pairs of OSCC and mucosal tissues was detected using ISH. *P < 0.05, **P < 0.01, ****P < 0.0001.

Through PCA (**Figure 2A**), 12 groups of data from GSE9844 dataset were used for differential expression analysis (GSM248652, GSM248655, GSM248658, GSM248660, GSM248687, GSM248780, GSM248665, GSM248668, GSM248677, GSM248678, GSM248682, GSM248685). The results showed that FENDRR is low expressed in OSCC tissues (**Figure 2B**). To further explore the expression of FENDRR in OSCC, we extracted the RNA of HIOECs, SCC4, SCC9, SCC25, CAL27, HN4, and 7 pairs of CAFs and NFs isolated from 7 OSCC patients. Interestingly, FENDRR is low expressed in HN4 and SCC25 compared to that in HIOECs, but highly expressed in SCC4 and CAL27 (**Figure 2C**). However, FENDRR is stably low expressed in CAFs compared to that in NFs (**Figure 2D**). Furthermore, the results of ISH indicated that FENDRR is mainly expressed in the stroma, and the expression level of FENDRR in the normal oral mucosal stroma is significantly higher than that in OSCC stroma (**Figures 2E, F**). Taking the lower expression level of FENDRR in OSCC tissues into account, we speculated that FENDRR may have a more significant effect on fibroblasts.

### FENDRR Inhibits the Proangiogenic Effect of CAFs

**Figure 3:**
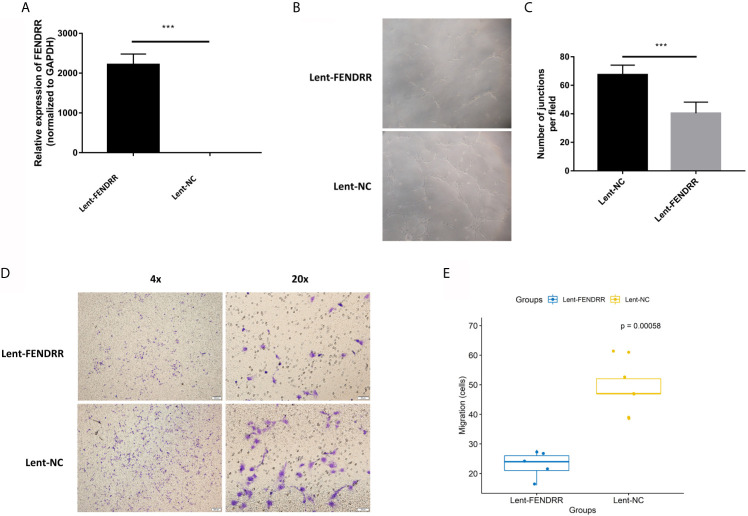
FENDRR inhibits the proangiogenic effect of CAFs. **(A)** FENDRR was significantly overexpressed in CAFs transfected with lent-FENDRR. **(B, C)** The number of the capillary-like structure formed by HUVECs co-cultured with CAFs transfected with Lent-FENDRR was significantly reduced. **(D, E)** The migration assay showed that CAFs transfected with Lent-FENDRR could inhibit the migration ability of HUVECs. ***P < 0.001.

Our previous studies confirmed that CAFs can promote the angiogenesis of melanoma (9). To explore the effect of FENDRR on the proangiogenic ability of OSCC-derived CAFs, we used lentiviral vectors to overexpress FENDRR in CAFs derived from OSCC. Real-time PCR was used to confirm the transfection effect. The results showed that FENDRR was significantly overexpressed in CAFs transfected with lent-FENDRR (**Figure 3A**). Subsequently, the CAFs transfected with Lent-FENDRR or Lent-NC were utilized to perform the migration and tube formation experiments. As shown in **Figures 3B–E**, the migration and tube-forming ability of HUVECs co-cultured with CAFs transfected with FENDRR was significantly suppressed, indicating that FENDRR can inhibit the proangiogenic ability of OSCC-derived CAFs.

### Survival Analysis and KEGG Analysis

**Figure 4:**
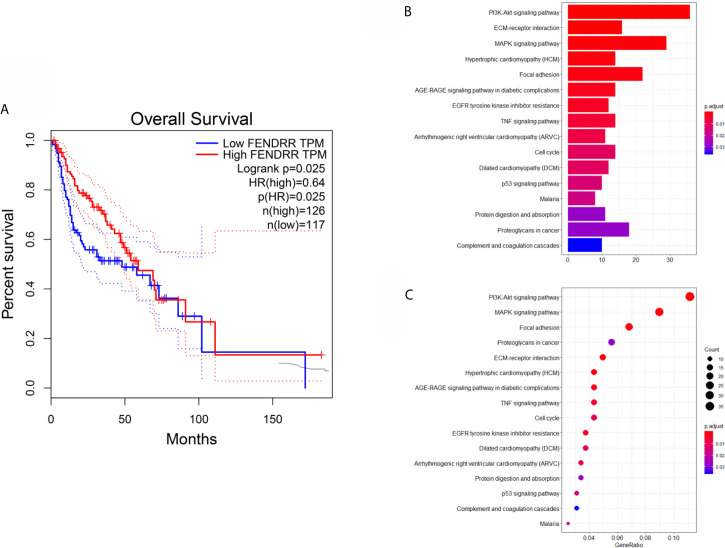
Survival analysis and KEGG analysis. **(A)** Survival analysis showed that HNSCC patients with high expression of FENDRR have a better prognosis. **(B, C)** KEGG analysis showed that genes differentially expressed between CAFs and NFs derived from HNSCC patients are mainly enriched to the PI3K/AKT pathway.

Angiogenesis is an essential factor leading to the low survival rate of OSCC patients (3, 4). Considering that FENDRR can weaken the angiogenesis of OSCC, we next performed the survival analysis of FENDRR using the GEPIA database. The results exhibited that HNSCC patients with high expression of FENDRR have a better prognosis (**Figure 4A**), which can further indicate that FENDRR may influence the progress of OSCC mainly by regulating fibroblasts. To further explore the possible effects of FENDRR on CAFs, we used the HNSCC dataset GSE83314, which contains two pairs of OSCC-derived CAFs and NFs, for differential expression analysis, and the differentially expressed genes were subsequently utilized for KEGG analysis. The results showed that genes differentially expressed between CAFs and NFs of HNSCC patients are mainly enriched to the PI3K/AKT pathway (**Figures 4B, C**).

### FENDRR Inhibits the Activation of the PI3K/AKT Pathway

**Figure 5:**
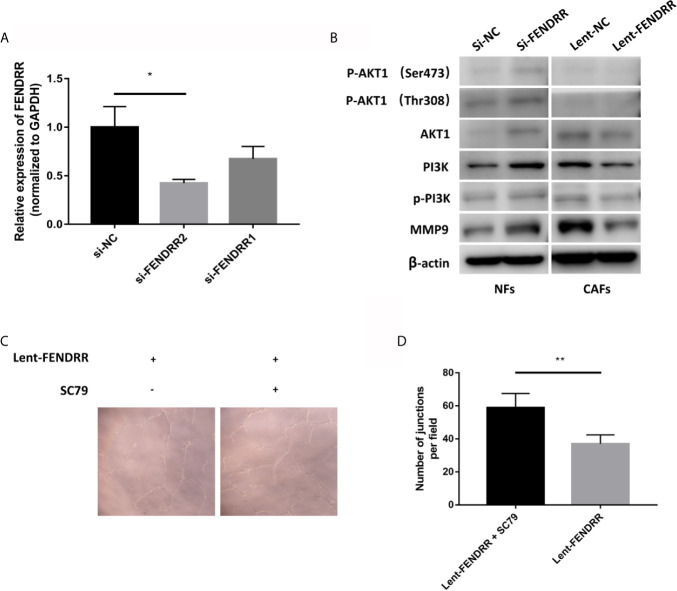
FENDRR inhibits the activation of the PI3K/AKT pathway. **(A)** The silencing effect of siRNAs. **(B)** Western blotting analyses of PI3K/AKT pathway-related genes showed that the knockdown of FENDRR activated the PI3K/AKT pathway in NFs, while the overexpression of FENDRR weakened the activation of the PI3K/AKT pathway in CAFs. **(C, D)** The number of the capillary-like structure formed by HUVECs co-cultured with SC79-treated CAFs transfected with Lent-FENDRR increased significantly. *P < 0.05, **P < 0.01.

Our previous study had demonstrated that the activation of the PI3K/AKT can promote the angiogenesis of OSCC (24). Herein, we continue to explore the influence of FENDRR on the PI3K/AKT pathway in CAFs. On the one hand, we used siRNAs to reduce the expression of FENDRR in NFs. On the other hand, lent-FENDRR and its negative control were used to overexpress FENDRR in CAFs. As shown in **Figure 5A**, the si-FENDRR2 has a better silencing effect than si-FENDRR1, so we chose si-FENDRR2 for subsequent knockdown experiments. Our results showed that the downregulation of FENDRR can activate the PI3K/AKT pathway in NFs and enhance the expression of MMP9. Consistently, the overexpression of FENDRR had opposite effects in CAFs (**Figure 5B**).

### FENDRR Inhibits the Proangiogenic Effect of CAFs Through the PI3K/AKT Pathway

To further explore whether FENDRR regulates the proangiogenic ability of CAFs through the PI3K/AKT pathway, we used SC79, a selective AKT activator (25), to activate the PI3K/AKT pathway in CAFs overexpressing FENDRR derived from OSCC, which then were used to perform the tube formation experiments. As shown in **Figures 5C, D**, the tube-forming ability of HUVECs co-cultured with SC79-treated CAFs transfected with FENDRR was significantly enhanced, indicating that FENDRR may inhibit the proangiogenic ability of OSCC-derived CAFs through the PI3K/AKT pathway.

### CAFs Overexpressing FENDRR Inhibit OSCC Progression *In Vivo*

**Figure 6:**
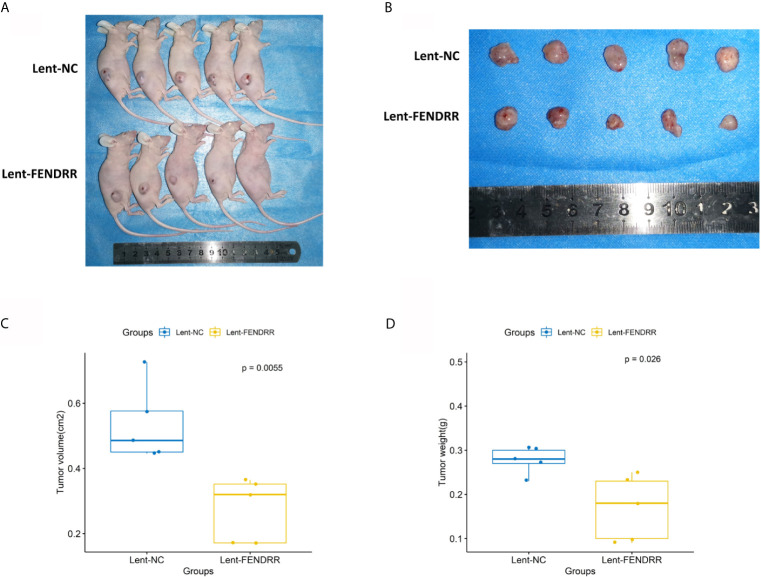
Overexpression of FENDRR in CAFs inhibits the progression of OSCC *in vivo*. **(A)** Images of nude mice from the Lent-NC and Lent-FENDRR groups. **(B)** Xenograft tumors excised from nude mice. **(C)** Volumes of the xenograft tumors excised from nude mice. **(D)** Weights of the xenograft tumors excised from nude mice.

To investigate the impact of FENDRR in CAFs on the progression of OSCC, the nude mice were divided into Lent-NC and Lent-FENDRR groups. CAL27 cells were mixed with CAFs transfected with Lent-NC and Lent-FENDRR respectively, and subcutaneously injected into nude mice. On the 23rd day, the tumors were excised and analyzed. The volume and weight of tumors in the Lent-FENDRR group significantly decreased compared with those in the Lent-NC group (**Figures 6A–D**). *In vivo* results showed that the high expression of FENDRR in CAFs can inhibit the progression of OSCC.

## Discussion

LncRNAs have been illustrated to play an important role in regulating the angiogenesis of various cancers (12–14). For example, Wang et al. reported that lncRNA HITT (HIF-1α inhibitor at translation level) can inhibit the angiogenesis of colon cancer by reducing the translation of HIF-1α in cancer cells (12). Besides, the proangiogenic effect of CAFs has also been confirmed by a large number of experiments (9, 10). However, there is currently no research involving the regulation of CAFs on the angiogenesis of OSCC mediated by lncRNAs. Therefore, in this research, we studied the role of FENDRR in the proangiogenic effect of CAFs derived from OSCC.

FENDRR is a long noncoding RNA located on chr3q13.31. Studies have shown that FENDRR is low expressed in a variety of tumor tissues and can inhibit tumor progression (26, 27). However, the role of FENDRR in the development of OSCC is still unknown. By analyzing high-throughput data, we found that FENDRR is low expressed in OSCC tissues compared with normal tissues. Furthermore, survival analysis showed that the low expression of FENDRR is related to the poor prognosis of HNSCC patients. Given that OSCC is the most common type of HNSCC (1), we speculated that FENDRR may also affect the progress of OSCC. Thus, we used Real-Time PCR to detect the expression of FENDRR in CAL27 and HIOECs, as well as 7 pairs of CAFs and NFs isolated from tissue samples of 7 OSCC patients. Interestingly, our results showed that FENDRR is low expressed in HN4 and SCC25 compared to that in HIOECs, but highly expressed in SCC4 and CAL27. However, FENDRR is stably low expressed in CAFs compared to that in NFs. Furthermore, the results of ISH indicated that FENDRR was mainly expressed in the stroma, and the expression level of FENDRR in the normal oral mucosal stroma was significantly higher than that in the OSCC stroma. Considering that the overall expression level of FENDRR in HNSCC tissues is decreased and patients with high FENDRR expression have a better prognosis, we speculated that FENDRR may influence the progress of OSCC mainly by regulating fibroblasts.

In our previous study, it had been shown that CAFs can facilitate melanoma angiogenesis, and the expression of the angiogenic factor MMP9 in CAFs is upregulated compared with that in NFs (9). Research by Li et al. confirmed that CAFs derived from OSCC can also promote the tube-forming ability of HUVECs (28). However, the role of lncRNAs in the proangiogenic effect of OSCC-derived CAFs is still unclear. In our study, the proangiogenic ability of CAFs overexpressing FENDRR was significantly reduced, and whether it was knockdown or overexpression of FENDRR, the expression level of MMP9 was opposite to that of FENDRR, which indicated that FENDRR may play an important role in the angiogenesis of OSCC regulated by CAFs.

MMP9 is an important angiogenic factor that has been proved to be regulated by multiple signaling pathways, such as PI3K/AKT pathway (29), ERK pathway (30), and JAK/STAT pathway (31). On the one hand, the activation of the PI3K/AKT pathway can enhance the mRNA and protein expression of MMP9 (29). On the other hand, our previous study demonstrated that the activation of the PI3K/AKT can promote the angiogenesis of OSCC (24). Herein, our results showed that the overexpression of FENDRR inhibited the PI3K/AKT signaling pathway in CAFs and decreased the expression level of MMP9. In addition, activation of the AKT pathway of CAFs overexpressing FENDRR can weaken the inhibitory effect of FENDRR on angiogenesis, which suggested that FENDRR may regulate the proangiogenic effect of CAFs derived from OSCC through the PI3K/AKT pathway.

At present, the specific mechanism by which FENDRR regulates the PI3K/AKT pathway is still unclear, and how CAFs overexpressing FENDRR affect the angiogenic ability of HUVECs still needs to be further explored.

## Conclusions

In general, our research has initially verified that lncRNA FENDRR regulates the proangiogenic ability of CAFs derived from OSCC through the PI3K/AKT pathway, which provides a theoretical basis for subsequent research.

## Data Availability Statement

The raw data supporting the conclusions of this article will be made available by the authors, without undue reservation.

## Ethics Statement

The animal study was reviewed and approved by the Review Board of the Ethics Committee of the Hospital of Stomatology at Wuhan University.

## Author Contributions

YX and EJ: conceived and designed the experiments, and contributed equally to this work. YX: analyzed the data and drafted the manuscript. ZJS and ZS: contributed to experimental studies and revised the manuscript. All authors contributed to the article and approved the submitted version.

## Funding

This study was supported by grants from the National Natural Science Foundation of China 81772897, 81672666, and the Natural Science Foundation of Hubei Province 2019CFB503.

## Conflict of Interest

The authors declare that the research was conducted in the absence of any commercial or financial relationships that could be construed as a potential conflict of interest.
